# Time course of EEG oscillations during repeated listening of a well-known aria

**DOI:** 10.3389/fnhum.2015.00401

**Published:** 2015-07-20

**Authors:** Lutz Jäncke, Jürg Kühnis, Lars Rogenmoser, Stefan Elmer

**Affiliations:** ^1^Division Neuropsychology, Institute of Psychology, University of Zurich, ZurichSwitzerland; ^2^International Normal Aging and Plasticity Imaging Center, University of Zurich, ZurichSwitzerland; ^3^Center for Integrative Human Physiology, University of Zurich, ZurichSwitzerland; ^4^University Research Priority Program, Dynamic of Healthy Aging, University of Zurich, ZurichSwitzerland; ^5^Department of Special Education, King Abdulaziz University, JeddahSaudi Arabia; ^6^Neuroimaging and Stroke Recovery Laboratory, Department of Neurology, Beth Israel Deaconess Medical Center and Harvard Medical School, Boston, MAUSA

**Keywords:** EEG oscillation, EEG time course, music, brain signature of music listening, heart rate, electrodermal response

## Abstract

While previous studies have analyzed mean neurophysiological responses to musical stimuli, the current study aimed to identify specific time courses of electroencephalography (EEG) oscillations, which are associated with dynamic changes in the acoustic features of the musical stimulus. In addition, we were interested in whether these time courses change during a repeated presentation of the same musical piece. A total of 16 subjects repeatedly listened to the well-known aria “Nessun dorma,” sung by Paul Potts, while continuous 128-channel EEG and heart rate, as well as electrodermal responses, were recorded. The time courses for the EEG oscillations were calculated using a time resolution of 1 second for several frequency bands, on the basis of individual alpha-peak frequencies (theta, low alpha-1, low alpha-2, upper alpha, and beta). For all frequency bands, we identified a more or less continuous increase in power relative to a baseline period, indicating strong event-related synchronization (ERS) during music listening. The ERS time courses, however, did not correlate strongly with the time courses of the acoustic features of the aria. In addition, we did not observe changes in EEG oscillations after repeated presentation of the same musical piece. Aside from this distinctive feature, we identified a remarkable variability in EEG oscillations, both within and between the repeated presentations of the aria. We interpret the continuous increase in ERS observed in all frequency bands during music listening as an indicator of a particular neurophysiological and psychological state evoked by music listening. We suggest that this state is characterized by increased internal attention (accompanied by reduced external attention), increased inhibition of brain networks not involved in the generation of this internal state, the maintenance of a particular level of general alertness, and a type of brain state that can be described as “mind wandering.” The overall state can be categorized as a psychological process that may be seen as a “drawing in” to the musical piece. However, this state is not stable and varies considerably throughout the music listening session and across subjects. Most important, however, is the finding that the neurophysiological activations occurring during music listening are dynamic and not stationary.

## Introduction

Music listening is a ubiquitous phenomenon in most people’s everyday life. The reason for this frequent usage of music in everyday life situations is that music is an extremely powerful stimulus for evoking and regulating emotions (Thoma et al., 2014). Therefore, it comes as no surprise that music is also often used in psychological and neuroscientific experiments as a stimulus for evoking emotions.

A wealth of studies have been published regarding the neurophysiological and physiological responses to emotional music (Koelsch, 2014). In such studies, many neuroscientific methods have been used to examine brain function during music listening, including functional magnetic resonance imaging (fMRI), positron-emission tomography (PET), magnetoencephalography (MEG), electroencephalography (EEG), transcranial magnetic stimulation (TMS), and even near-infrared spectroscopy (NIRS). In general, these studies have shown that the limbic system, as well as the cortical areas, are strongly activated when listening to emotionally arousing music. One important region is the nucleus accumbens, which is activated by listening to pleasant music, while the amygdala is strongly activated when listening to unpleasant or sad music (Blood et al., 1999; Baumgartner et al., 2006b; Alluri et al., 2013). Many other cortical regions besides the limbic system have also been shown to be involved in processing emotional music. Depending on the method used to register brain activations, different distributed cortical networks have been identified, which cover numerous other regions in the frontal, temporal, and parietal lobes (Koelsch, 2014).

Nearly all of the studies on brain activation in response to emotional music have analyzed mean neurophysiological and psychological responses that last from seconds to several minutes. For example, average hemodynamic or electrophysiological responses to musical pieces are usually used for interpretation (Blood et al., 1999; Blood and Zatorre, 2001; Baumgartner et al., 2006b; Koelsch et al., 2007, 2013; Sammler et al., 2007). The same pertains to the subjective evaluation of musical pieces. Typically, study participants are asked to rate entire musical pieces using various forms of rating scales for valence and arousal, or other subjective variables such as the tension evoked by the piece. These ratings are typically employed as though the musical experience were a stationary phenomenon. However, music is a dynamic stimulus, with changing acoustic features across time. Thus, the neurophysiological and psychological responses should vary according to the changing acoustic cues of the musical piece. Using continuous ratings of a musical piece, it has already been demonstrated that subjective ratings indeed vary during the course of music listening (Nagel et al., 2007). This idea especially holds for longer musical pieces, like symphonies or operas, which are designed to evoke changing states of emotion and arousal. Even short musical pieces from the modern pop genre, which last approximately 5 min, include changing acoustic features over the music’s presentation.

Taking this information into account, it can hardly be expected that music would evoke a constant psychological and neurophysiological reaction, even within one single subject. Instead, these psychological and neurophysiological responses should change during the course of music presentation. In addition, listening repeatedly to a particular musical piece may evoke different responses, either due to the listener taking different approaches to listening to the music or he/she habituating to repeated exposure to the stimuli. A further issue that should also be kept in mind in this context is that the psychological and neurophysiological responses to musical pieces will vary greatly according to the actual psychological state of the subject and according to the “strategy” used when listening to the particular musical piece.

Interestingly, although the above-mentioned aspects seem to be plausible and are grounded in long-existing knowledge about how we perceive musical pieces, it is surprising that so few psychological and neurophysiological studies have examined time courses of neurophysiological activation during music listening. One reason for the apparent lack of literature in this area is related to methodological issues. fMRI measurements during music listening are particularly problematic due to the obtrusive scanner noise that affects activations in the auditory system (Herrmann et al., 2000). Another problematic point for music and emotion studies using fMRI is the uncomfortable scanner environment, which is associated with negative emotions such as discomfort, claustrophobia, pain, low-level anxiety, and other variants of negative emotion (Heinrich et al., 2014; Mutschler et al., 2014; Keulers et al., 2015). In addition, the measured hemodynamic responses to music are slow and inert, allowing a time resolution in the range of only 4–8 s, at best.

Chapin et al. (2010) did, however, use fMRI to investigate the neural correlates of emotional arousal during a Chopin étude. They correlated the hemodynamic responses to this musical piece with the arousal ratings obtained during music presentation and identified several brain areas (middle occipital gyrus, dorsal anterior cingulate, middle frontal gyrus, and medial frontal gyrus), for which the hemodynamic responses were associated with the arousal rating. Lehne et al. (2014) employed a similar approach, relating subjectively experienced tension ratings during piano pieces to hemodynamic responses. They identified hemodynamic responses in the left lateral orbitofrontal cortex, and to a lesser extent in the amygdala, that were related to increased or decreased subjectively experienced tension.

Other studies have used PET to study blood flow responses to musical stimuli (Satoh et al., 2006). Although PET measurements are silent, which is beneficial when measuring neurophysiological responses to auditory stimuli, these measurements are unfortunately associated with injections of tracers into the blood of the subjects, which is a stressful intervention for many subjects. In addition, the time resolution for blood flow measured with PET is even lower than for the hemodynamic responses measured with fMRI, ranging from several seconds to minutes. Thus, this measure is actually not very well suited for time series analyses. EEG and MEG, on the other hand, measure neurophysiological responses on the millisecond scale and are thus much better suited for analyzing time courses of neurophysiological reactions to musical stimuli. A further advantage of EEG is that it is easily available, relatively inexpensive, and can be used in an ecologically valid setting (e.g., sitting on a chair while listening to hi-fi music without any interfering noise). In addition, although EEG signals are prone to contamination by muscle and eye-movement artifacts that hamper time series analysis, several mathematical correction methods are available that make it possible to subtract artifacts from EEG signals, thus allowing the analysis of an (more or less) artifact-free, continuous EEG signal.

To the best of our knowledge, only two published studies have analyzed the time course of EEG responses during music listening and related these time courses to subjective emotional responses (Mikutta et al., 2012, 2014). Mikutta et al. (2012, 2014) analyzed the EEG activations of study participants while listening to Beethoven’s fifth symphony, a musical piece that lasts 442 s. For each second, they computed the power in different frequency bands and related these measures to subjective and continuous arousal ratings. The latter ratings were obtained in a second trial, in which the subjects listened again to the same symphony without electrophysiological recordings, in order to prevent contamination of the EEG signals by movement-related neurophysiological activations. Using this approach, the authors identified a right-frontal suppression of lower alpha-band activity during periods of high subjective arousal. This finding is consistent with the idea that right-sided frontal brain regions are involved in the control of arousal (Meadows and Kaplan, 1994; Nitschke et al., 1999; Craig, 2005). It has been suggested that this lateralization is due to asymmetrical input from the autonomic nervous system, in which the right hemisphere receives preferentially sympathetic input that is associated with arousal, orientation responses, and negative affect. The left hemisphere receives mostly parasympathetic input, occurring predominantly during states of low arousal and positive affect.

Nevertheless, although the pioneering described above work has paved the way for the analysis of the EEG activity time course during music listening, some issues still remain unanswered. For example, Mikutta et al. (2012, 2014) reported time courses for subjective arousal ratings obtained during a second run, during which no EEG recordings were made. During the music presentation, participants were asked to rate their subjective arousal via mouse movements. In particular, they were asked to move their mouse forward when they felt a heightened inner arousal that was independent of their affective valence. When they felt reduced arousal, they were to move the mouse backward. In this way, the participants provided a continuous rating of their subjective experience of arousal. However, it is not certain whether the subjective arousal experience indicated during the second music presentation was similar to the arousal experienced during the first music presentation, when no mouse movements were required. Secondly, this arousal rating is a reactive and consciously driven rating: the subjects must decide to manipulate the mouse to indicate their arousal level. Such a requirement evokes introspection, which is associated with particular brain activations (Hutcherson et al., 2005). A further unexplored aspect is whether the psychological, physiological, and neurophysiological responses differ between repeated presentations of the same music. The subjects may have used a different listening approach during the second music presentation than during the first. Some habituation might also have taken place during the second music presentation.

Building on these initial studies of the time sequence of brain activity during music presentation, we used EEG to measure neurophysiological activation over time and evaluated the time courses of the theta, alpha, and beta frequency bands corrected for muscle and eye movement artifacts. In order to test whether these neurophysiological fluctuations differ across music presentations, we repeatedly presented one particular musical piece and tested whether a type of habituation takes place as a function of repeated presentation. Secondly, we analyzed the time course of electrodermal and heart responses during the course of the music presentation. These psychophysiological responses were used as indicators of physiological arousal; they are also known to be valid indicators of subjective experienced arousal (Baumgartner et al., 2006a; Nater et al., 2006; Mikutta et al., 2013).

We addressed the following research questions:

(1) Is the time course of neurophysiological activation related to the time sequences of the acoustic features of the musical piece? Here, we focus on intensity envelope and acoustic complexity.
(2) Is the time course of neurophysiological activation related to the time course of psychophysiological arousal? Here, we use electrodermal activity (EDA) and heart rate (HR) as indicators of psychophysiological arousal.
(3) How stable is the time course of neurophysiological activation over repeated presentations of the same musical stimulus?
(4) Similarly, do EDA and HR during music listening change over repeated exposure to the same musical piece?

## Materials and Methods

### Subjects

Sixteen students (4 men, mean age = 23.5 years, SD = 2.8 years, and 12 women, mean age = 22.2 years, SD = 8.3 years, all native German speakers) enrolled in psychology classes participated in the present study. All subjects were consistently right-handed, as revealed by the Edinburgh Handedness Inventory (Oldfield, 1971), except for one subject who was classified as moderate right-hander. In order to control for general cognitive abilities, we applied two short intelligence tests, namely the KAI (Kurztest der aktuellen geistigen Leistungsfähigkeit; Lehrl et al., 1992) and the MWT (Mehrfachwahl-Wortschatz-Intelligenz; Lehrl et al., 1974; Hessler et al., 2013). These tests revealed above average general cognitive abilities for the participating subjects (KAI-IQ: mean = 133.6, SD = 10.06; MWT-IQ: mean = 110.13, SD = 9.6). The musical aptitudes of the participants were estimated using the “Advanced Measure of Music Audiation” (AMMA) test published by Gordon (1989). This procedure is based on the assumption that a fundamental prerequisite for musical aptitude is the ability to hold musical sounds in memory and detect melodic and rhythmic variations. During the AMMA test, the volunteers listened to short pairs of piano tone sequences and had to decide whether these sequences were equivalent, rhythmically different, or tonally different. The subjects scored above average (according to the norms of the Gordon test; total score for the subject sample of this study: 69.1, SD = 21.5; mean and SD of the norm population for non-musicians = 50.6 ± 7.9). No subjects reported a history of present or past neurological, psychiatric, or audiological disorders, and all possessed an unremarkable audiological status, as revealed by pure tone audiometry (Home Audiometer software^1^). All subjects denied consuming illegal drugs or regular medication. The subjects were paid for their participation, the local ethics committee approved the study, and written informed consent was obtained from all subjects. None of the subjects indicated any history of musical training, as assessed by an in-house questionnaire frequently used by our research group.

### Stimulus

**FIGURE 1:**
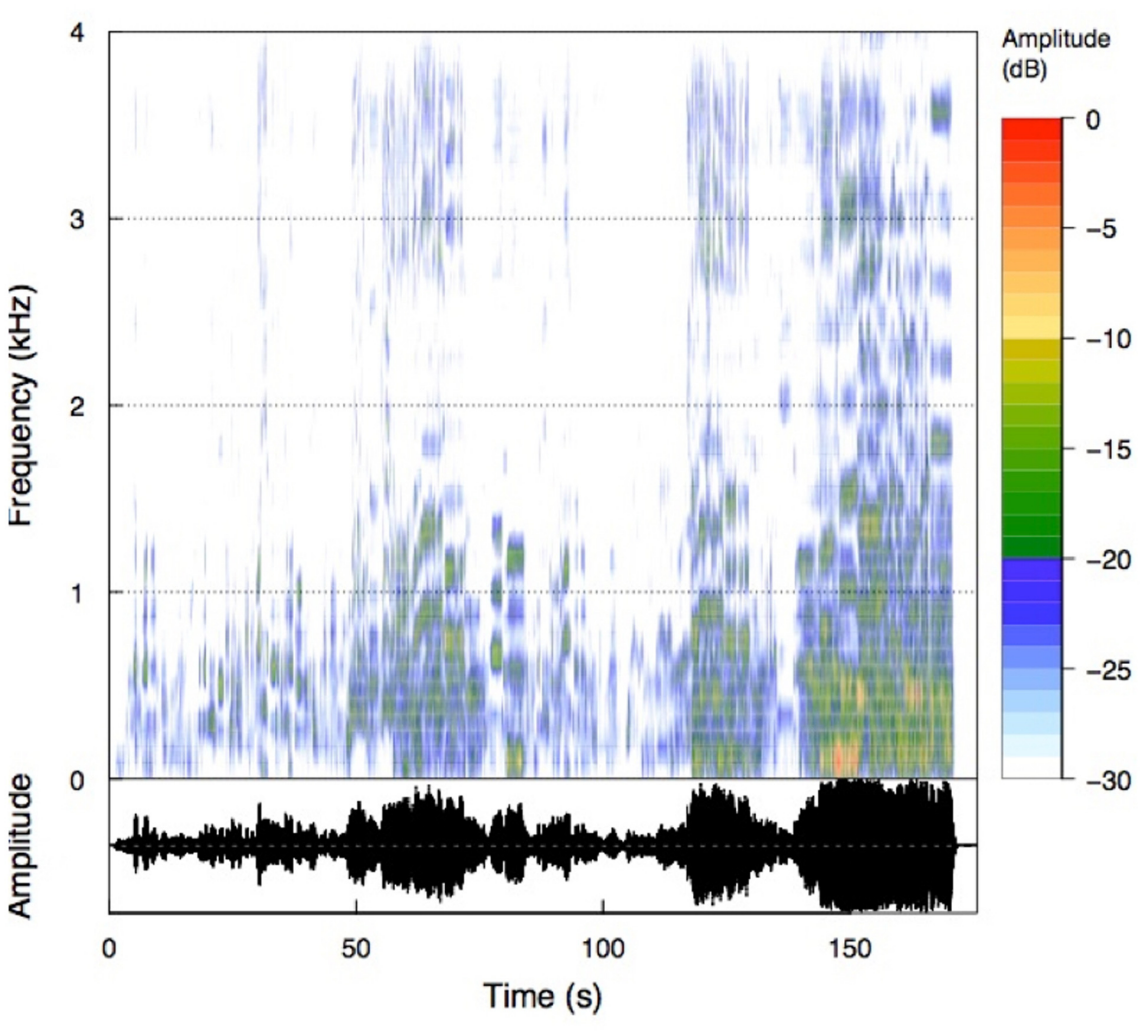
**Time frequency analysis of the musical piece (Nessun dorma).** The spectrogram is scaled relative to the maximum intensity of the musical piece.

We used the well-known aria “Nessun dorma,” sung by Paul Potts, as the music stimulus. This musical piece was chosen because it evokes strong emotions, is well known, and has a strong dynamic acoustic range (see **Figure 1**). The duration of the musical piece is about 175 s, or approximately 3 min. The relatively short duration of this aria was an added benefit, as we were interested in studying the neurophysiological and psychophysiological responses to repeated presentation of the stimulus. We used the iTunes^©^ version of the song and transformed the mp4 format into the widely used mp3 format. The musical stimulus was presented via HiFi earphones (Sennheiser, CX-350, Colchester, Essex, UK) in convenient loudness (intensity = 75 dB). The time–frequency histogram of the music stimulus is presented in **Figure 1**. As can be seen from the amplitude time course of the musical piece, the aria starts with low intensity and from there continues to increase in intensity. This is nicely depicted in the rectified amplitude time course (AMP) shown in **Figure 4**. Although some non-linear trends are evident, there is also a clear linear trend in intensity, qualified by stronger intensities at the end of the aria.

### Procedure

**FIGURE 2:**
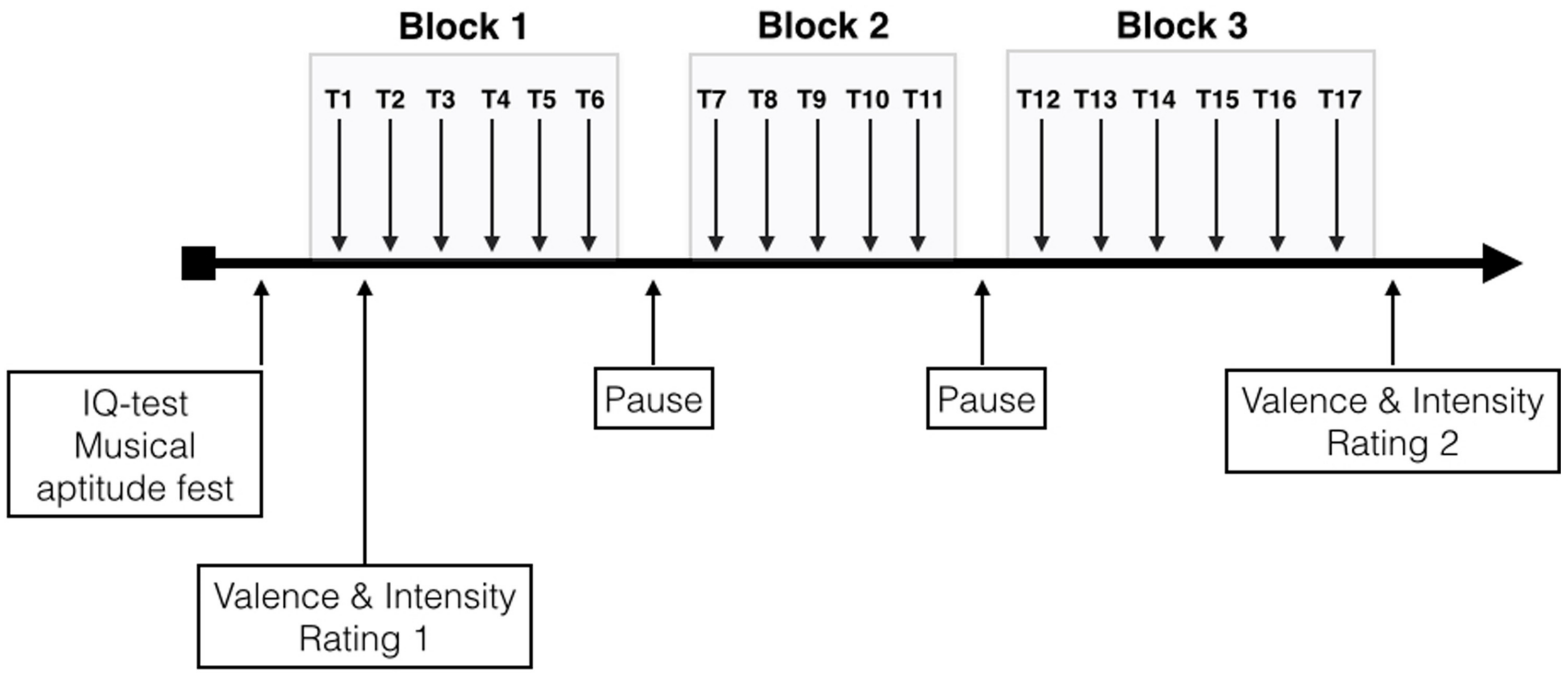
**Schematic description of the experimental design (T1–T17 are the different trials)**.

Over the entire course of the study, participants were seated in a comfortable chair in a sound-shielded room. The music stimulus was presented in three blocks. Block-1, Block-2, and Block-3 comprised six, five, and six repeated presentations of the music stimulus, respectively. Thus, the duration for Block-1 and Block-3 was 18 min, while it was 15 min for Block-2. Between each block, a pause of 5 min was given. Due to the pauses before and after Block-2 we shortened Block-2 in order to gain some time to control the EEG montage. During each pause, the experimenter ensured that electrode impedance remained low enough and applied some saline to any electrodes that showed increased impedance. After the first trial of Block-1 and the last trial of Block-3, the subjects rated the musical piece according to valence and arousal using analog scales based on the Self-Assessment Manikin (SAM) for valence and arousal as proposed by Bradley and Lang (1994). These ratings were done on a computer monitor placed in front of the subjects, on which they were shown a vertical line for valence and a horizontal line for arousal. The SAM for valence ranged from a frowning, unhappy figure (top) to a smiling, happy figure (bottom). The middle position was illustrated with a neutral figure. The subjects were instructed to click on the position of this vertical valence line that best represents their actual valence rating. The values for each position on the valence line ranged from -1000 (top = unhappy) to +1000 (happy). Thus, values > 0 point to positive valence while values < 0 to negative valence. For arousal rating we used a horizontally arranged analog scale ranging from a relaxed, sleepy figure to an excited, wide-eyed figure. This corresponding values ranged from 0 (leftmost = relaxed) to 1000 (rightmost = excited). The subjects first performed the valence rating, followed by the arousal rating. A schematic presentation of the experimental procedure is depicted in **Figure 2**. During the music presentation, EEG, EDA, and HR were recorded, and subjects were instructed to close their eyes and listen carefully to the music during the entire measurement period. The closed-eyes condition was chosen to minimize interference from visual input. Furthermore, subjects were asked not to clench their teeth and to avoid any kinds of movements.

### EEG Recording and Data Reduction

Electroencephalograms were recorded using a high-density Geodesic EEG system^^®^^ (GSN300; Electrical Geodesic Inc., Eugene, OR, USA) with a 128-Channel HydroCel Geodesic Sensor Nets@ (HCGSN120). Data was sampled at 500 Hz and band-pass filtered at 0.1–100 Hz. The vertex electrode (Cz) served as an on-line reference. Impedance was maintained below 30 kΩ. For the exact positioning of the onset of music in the EEG, a marker channel was used to indicate the start and end of the musical piece. EEG analysis was conducted to identify the spectral correlates of music-induced fluctuations in cortical activations. EEG data were analyzed with the Brain Vision Analyzer version 2.0.1 (Brain Products GmbH, D-82205 Gilching). In a first step, raw EEG data were band-pass filtered (1–40 Hz) including a notch-filter of 50 Hz to eliminate even very small oscillations leaking above 40 Hz. Eye movements and muscle artifacts were corrected by applying independent component analysis (Delorme et al., 2007). In addition, remaining muscle artifacts were identified and eliminated using ASR (The Artifact Subspace Reconstruction Method developed and programmed by Christian A. Kothe^2^^,^^3^), a new algorithm designed to remove non-stationary high-variance signals from EEG time series and reconstruct the missing data using a spatial mixing matrix (assuming volume conduction). Next, the data were parsed into 175 epochs, each 1 s in duration. All epochs and channels were recomputed to average reference and frequency transformed by means of a fast Fourier transform (FFT). Spectral amplitude (μV^2^/Hz) was then computed.

**FIGURE 3:**
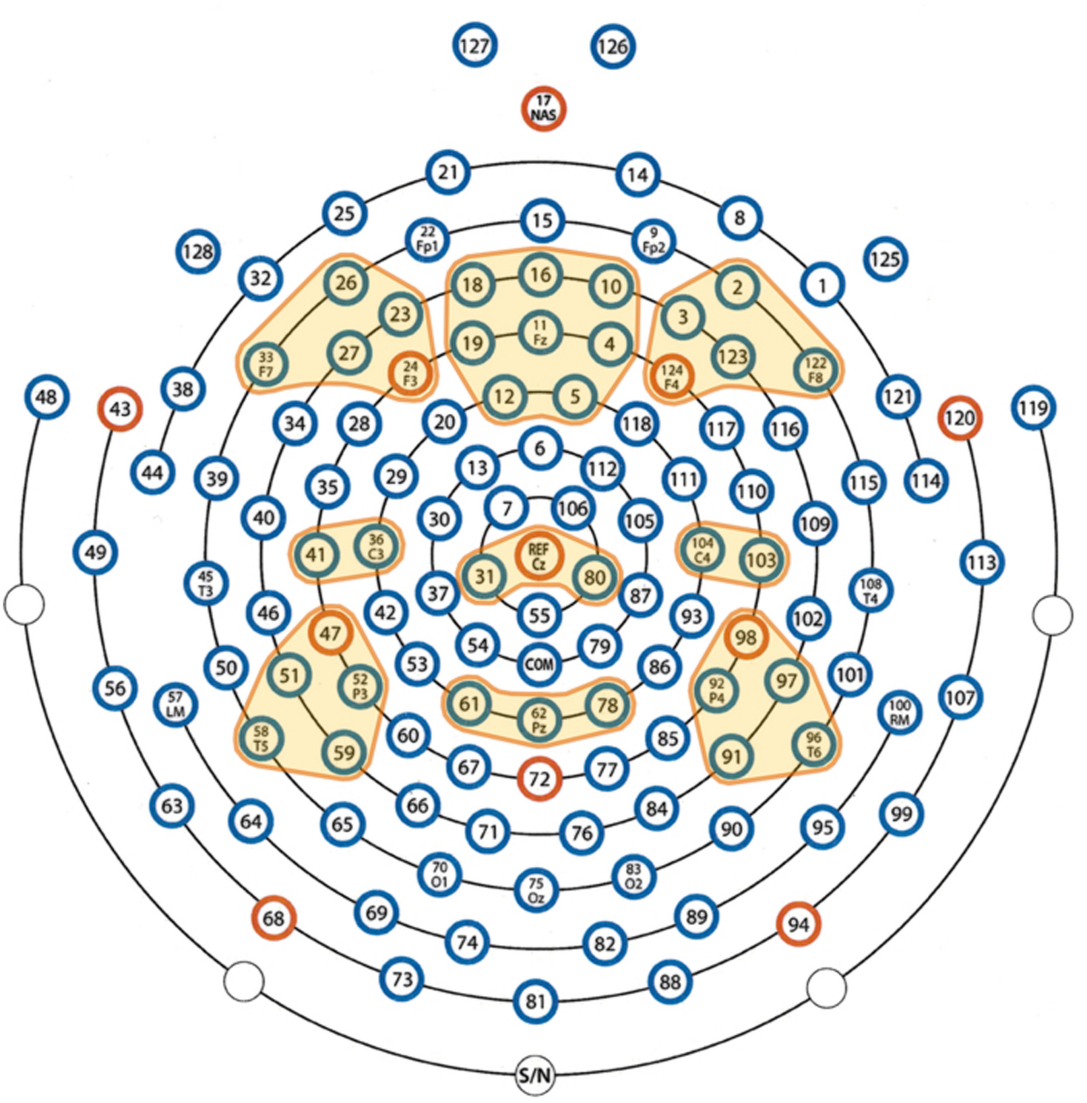
**Definition of the electrode clusters of interest (EOIs)**. The EOIs are color-coded in yellow.

For frequency analyses, we used the individual alpha frequency (IAF) as the reference frequency according to which all other frequency bands were determined. For this determination, we used the following individually adjusted frequency bands according to Doppelmayr et al. (1998), which were also used by Sammler et al. (2007): Theta: IAF × 0.4-IAF × 0.6; Lower alpha-1: IAF × 0.6 HZ-IAF × 0.8 HZ; Lower alpha-2: IAF × 0.8 HZ-IAF × 1.0 HZ; Upper alpha: IAF × 1.0-IAF × 1.2; Beta: IAF × 1.2-30 Hz. In order to gain statistical power, we defined nine electrode clusters of interest (EOI) for which the mean power of the frequency bands was calculated: three frontal, three central, and three parietal (left, midline, and right; the EOIs are shown in **Figure 3**). The EOIs comprised the following sensors: left frontal (LF) = 23 (F3), 24, 26, 27, and 33 (F7); midline frontal (MF) = 4, 5, 10, 11 (Fz), 12, 16, 18, and 19; right frontal (RF) = 2, 3, 122 (F8), 123, and 124 (F4); left central (LC) = 36 (C3) and 41; midline central (MC) = 31, Cz, and 80; right central (RC) = 103 and 104 (C4); left parietal (LP) = 47, 51, 52 (P3), 58, and 59; midline parietal (MP) = 61, 62 (Pz), and 78; right parietal (RP) = 91, 92 (P4), 96, 97, and 98 (for orientation please refer to the map in **Figure 3**). These EOIs were chosen because they symmetrically cover the frontal, central, and parietal scalp regions of both hemispheres. Of the EOI selection, F3, Fz, F4, C3, Cz, C4, P3, Pz, and P4 were chosen as pivotal and central electrodes. Applying Geodesics EEG montages, several papers have used similar or even the same electrodes of interest (e.g., Curran and Dien, 2003). In order to account for individual intrinsic electrophysiological differences, we calculated power values relative to a 5 s baseline of EEG obtained immediately before the start of the musical piece. These relative values are similar to the event-related desynchronization (ERD) and event-related synchronization (ERS) measures proposed by Pfurtscheller and Andrew (1999):

$$ERDorERS=\frac{\left(A-B\right)}{B}$$

where *B* is the mean power during the baseline session, and *A* represents the mean power during the stimulation period. In our study, the ERD/ERS values were calculated and subjected to statistical analyses. For the sake of completeness, we also computed global field power (GFP) across the frequency bands we have studied here (1–30 Hz), which is the SD of the momentary potential values across all electrodes (Lehmann and Skrandies, 1980)

Electrodermal activity and HR were recorded using a Biopack MP100 amplifier. To measure EDA, we placed two electrodes on the palm of the participant’s subdominant hand. For HR recording, we placed two electrodes on each inner forearm, as well as a reference electrode on the elbow of the subdominant arm. Data were sampled at 200 Hz. HR was automatically calculated online as beats per minute (bpm). For pre-processing, we segmented the data into 1 s segments, linearly detrended each segment, and related these values to the 4 s baseline prior to music presentation. Thus, our HR and EDA values represent differences in bpm (HR) and μMho relative to baseline.

**FIGURE 4:**
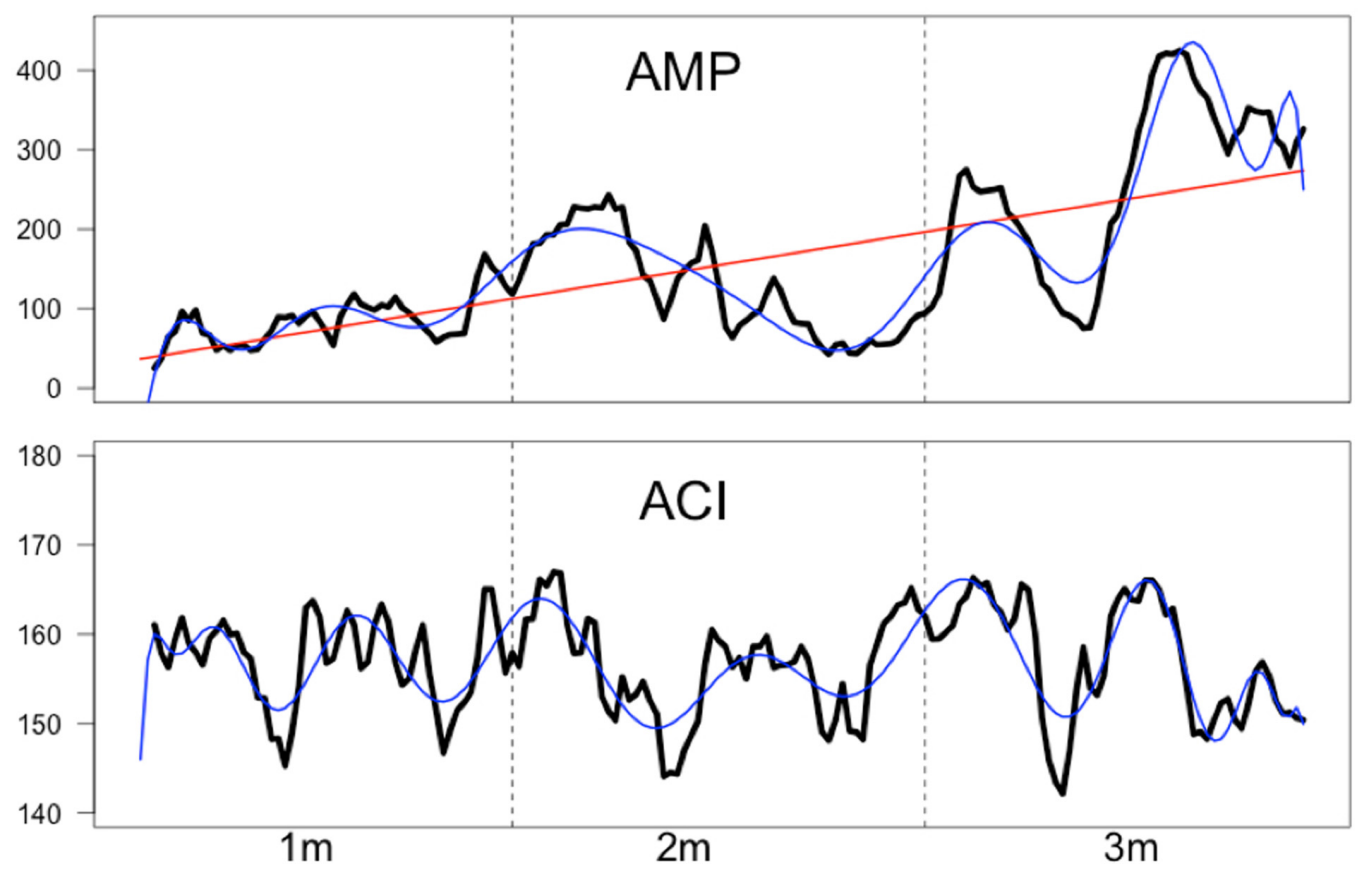
**Time series (in arbitrary values) of the rectified amplitude (AMP) and the acoustic complexity (ACI) of the aria.** The red line (for AMP) indicates a linear trend (*R*^2^ = 0.446) while the blue lines (for AMP and ACI) indicate higher order polynomials. A 20th order polynomial fitted to AMP nearly perfectly describes the AMP time course (*R*^2^ = 0.89). A 40th order polynomial fits the ACI time course very well (*R*^2^ = 0.64).

From the acoustic music stimulus, we extracted the intensity envelope using a root mean square rectification and integration across a time window of 1 s, resulting in 175 data points for the acoustic envelope (AMP). In addition, the acoustic complexity index (ACI) was computed separately for each second, also resulting in 175 time points for the ACI index. The ACI measure represents the complexity of the sound spectrogram and has been frequently used to analyze bird songs (Pieretti et al., 2011). ACI was computed using a subroutine of the R software package “seewave,” which is specifically designed to analyze sound information (Sueur et al., 2008). In short, ACI represents for a given time period the summed difference between the intensities of adjacent frequency bins. Thus, the ACI represent variations of intensity in each single frequency bin. The exact formula can be found in Pieretti et al. (2011). The time courses of these acoustic measures are shown in **Figure 4**. As can be seen from this figure the rectified amplitude of the musical stimulus increases throughout music presentation. Fitting a first order (linear) polynomial to this time course revealed a strong linear trend (*R*^2^ = 0.446). With a 20th order polynomial, this time series is nearly perfectly modeled (*R*^2^ = 0.89). For the ACI time course, a 40th order polynomial explains the time course quite well (*R*^2^ = 0.64). There was, however, no linear trend (*R*^2^ = 0.005).

In sum, we measured the time courses of the EEG ERD/ERS ratios for the different frequency bands (theta, low alpha-1, low alpha-2, upper alpha, and beta) and the different EOIs (LF, MF, RF, LC, MC, RC, LP, MP, and RP), the time courses of EDA and HR, and the time courses of AMP and ACI. All time courses were computed with a time resolution of 1 s, resulting in 175 time points and 17 repetitions corresponding to the 17 trials during which the musical stimulus was presented. In later processing steps we used different time resolutions (see below).

### Statistical Analysis

**FIGURE 5:**
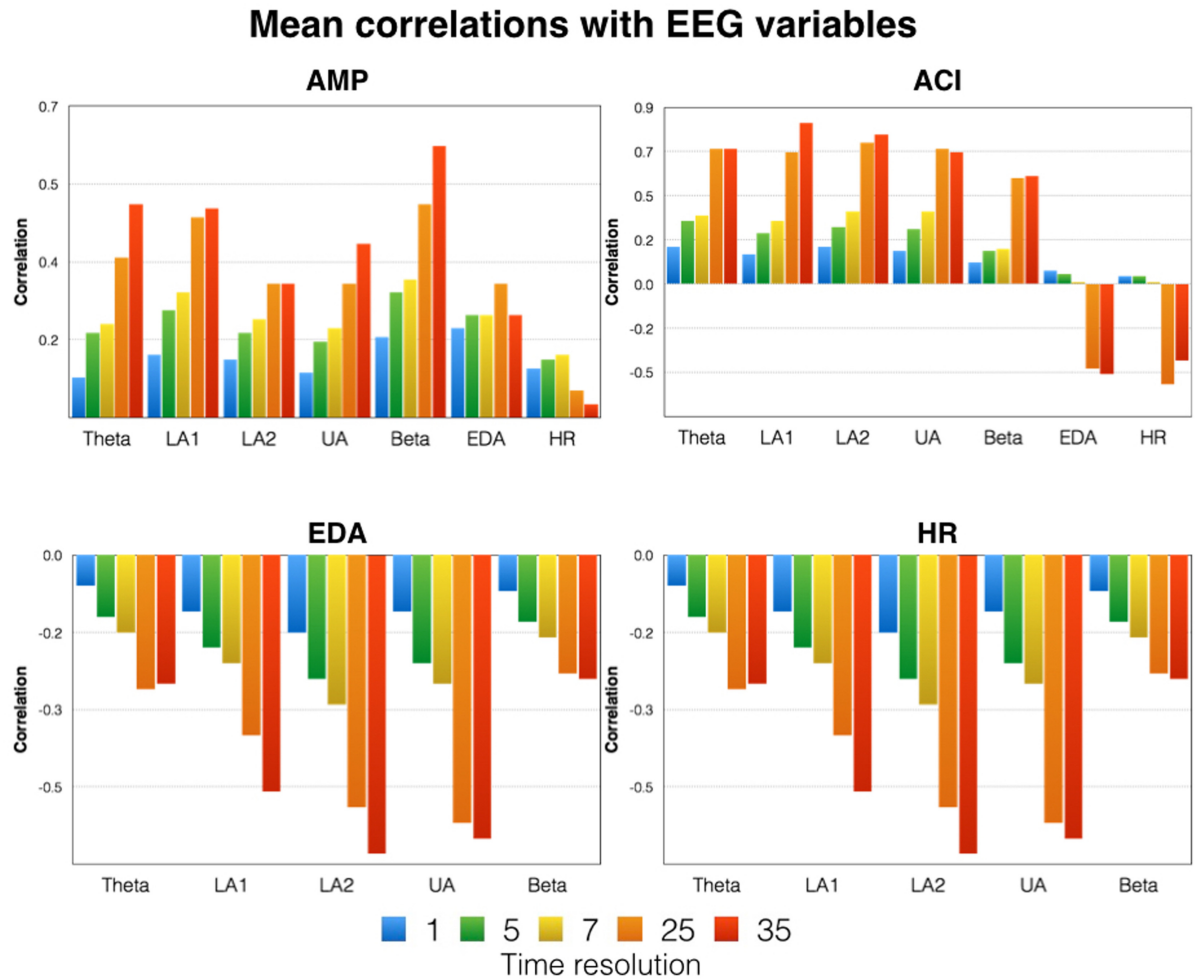
**Mean correlations between the time courses of the acoustic variables (AMP and ACI) and the physiological variables (first row).** The correlations between psychophysiological and neurophysiological variables are shown in the second row. Please note that the data shown are mean correlations obtained by averaging z-transformed correlations and re-transforming them into correlations. Indicated here are the correlations for five different time resolutions [175 time frames (1 s period indicated in blue) = 1 Hz, 35 time frames (5 s period indicated in green) = 0.2 Hz, 25 time frames (7 s period indicated in yellow) = 0.14 Hz, 7 time frames (25 s period indicated in orange) = 0.04 Hz, 5 time frames (35 s period indicated in red) = 0.028 Hz]. LA1, low alpha-1; LA2, low alpha-2; UA, upper alpha; EDA, electrodermal activity; HR, heart rate.

**FIGURE 6:**
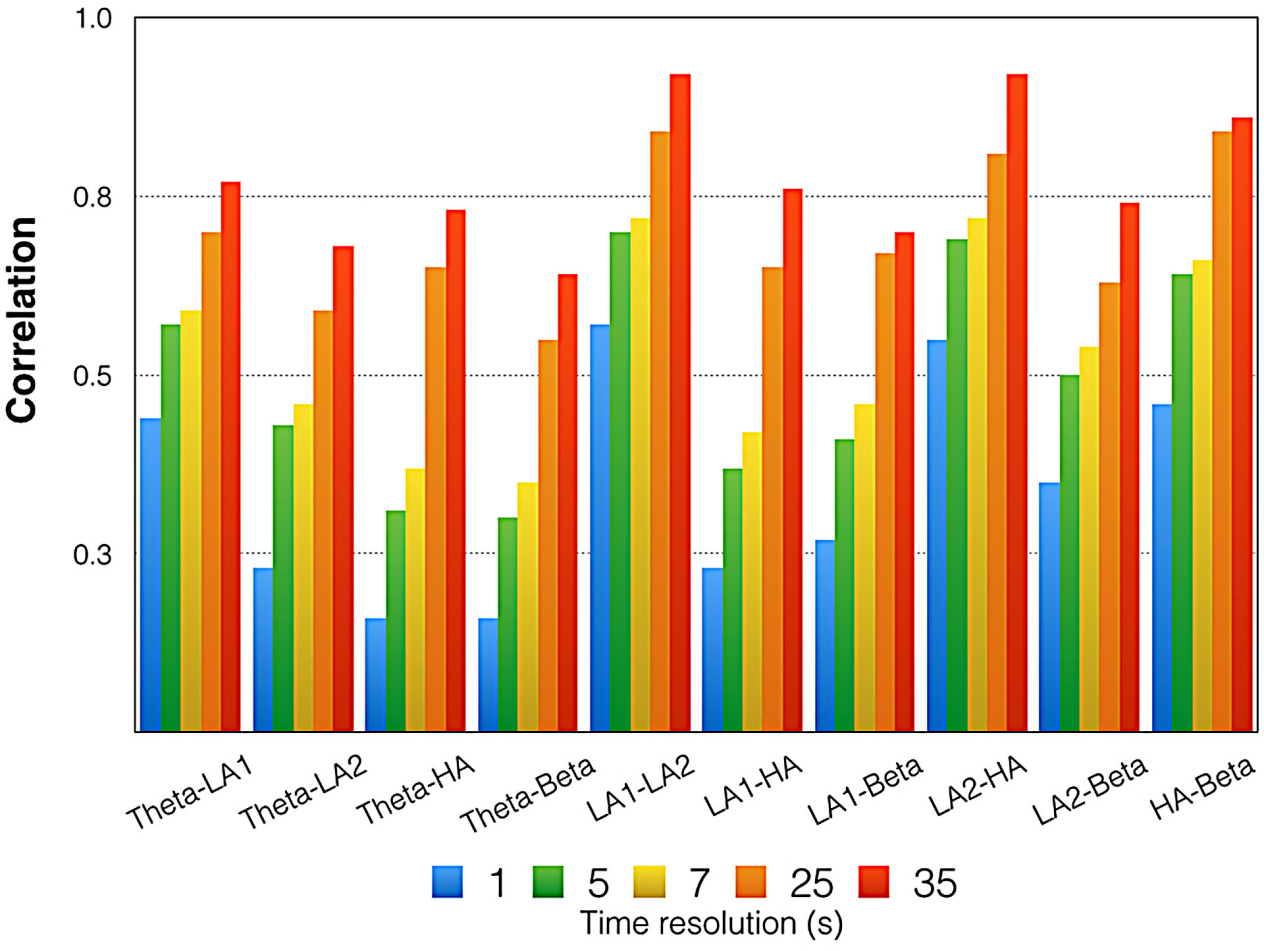
**Mean correlations between the time courses of all EEG oscillations.** Please note that these are mean correlations obtained by averaging z-transformed correlations and re-transforming them into regular correlations. Indicated here are the correlations for five different time resolutions [175 time frames (1 s period indicated in blue) = 1 Hz, 35 time frames (5 s period indicated in green) = 0.2 Hz, 25 time frames (7 s period indicated in yellow) = 0.14 Hz, 7 time frames (25 s period indicated in orange) = 0.04 Hz, 5 time frames (35 s period indicated in red) = 0.028 Hz]. LA1, low alpha-1; LA2, low alpha-2; UA, upper alpha.

**FIGURE 7:**
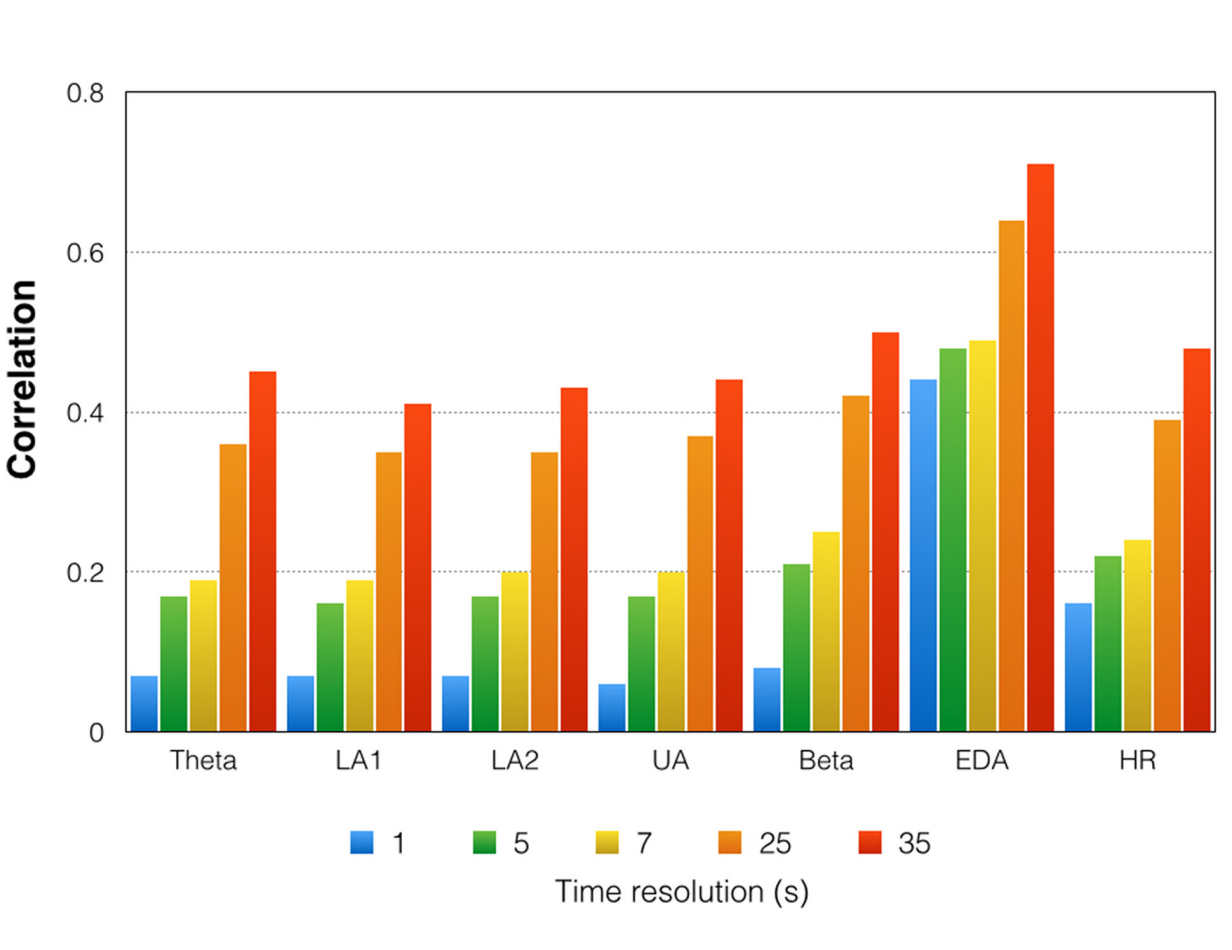
**Mean correlations between the 17 trials for the time courses of all physiological variables.** Please note that these are mean correlations obtained by averaging z-transformed correlations and re-transforming them into regular correlations. Indicated here are the correlations for five different time resolutions [175 time frames (1 s period indicated in blue) = 1 Hz, 35 time frames (5 s period indicated in green) = 0.2 Hz, 25 time frames (7 s period indicated in yellow) = 0.14 Hz, 7 time frames (25 s period indicated in orange) = 0.04 Hz, 5 time frames (35 s period indicated in red) = 0.028 Hz]. LA1, low alpha-1; LA2, low alpha-2; UA, upper alpha; EDA, electrodermal activity; HR, heart rate.

**FIGURE 8:**
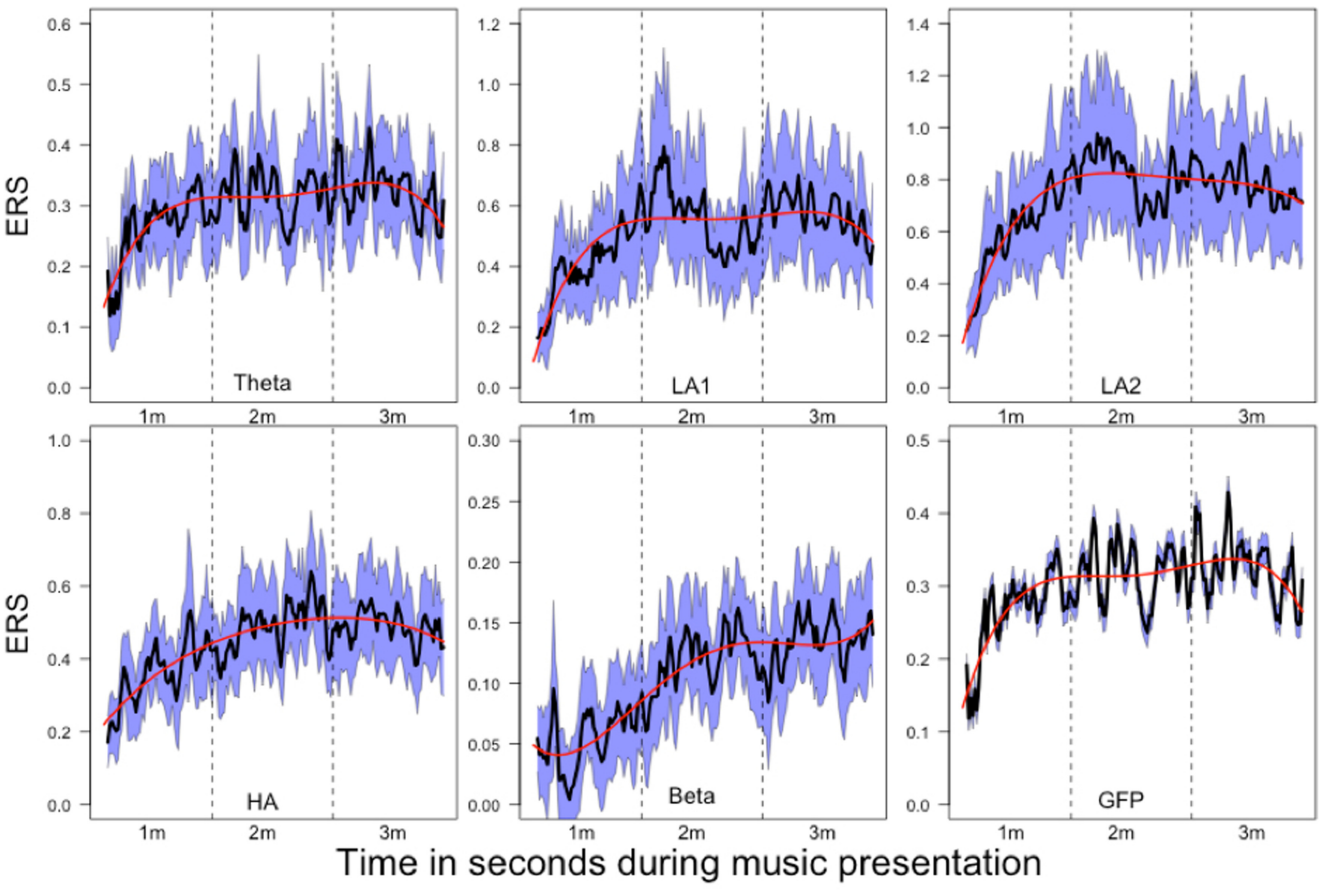
**Mean relative power changes (relative to baseline) for the frequency bands and global field power (GFP) during music listening**. Since the relative changes in power are positive, we have denoted them as event-related synchronization (ERS). The dashed vertical lines separate the time courses into segments of 1 min duration (1m: 1st minute, 2m: 2nd minute, 3m: 3rd minute). The SEM (calculated across subjects) are plotted as blue area around the mean (plotted as a black line). The red line indicates the 4th order polynomials fitted to these time courses. These fits explain the time courses very well (*R*^2^ ranging from *R*^2^ = 0.53 for GFP to *R*^2^ = 0.78 for beta).

**FIGURE 9:**
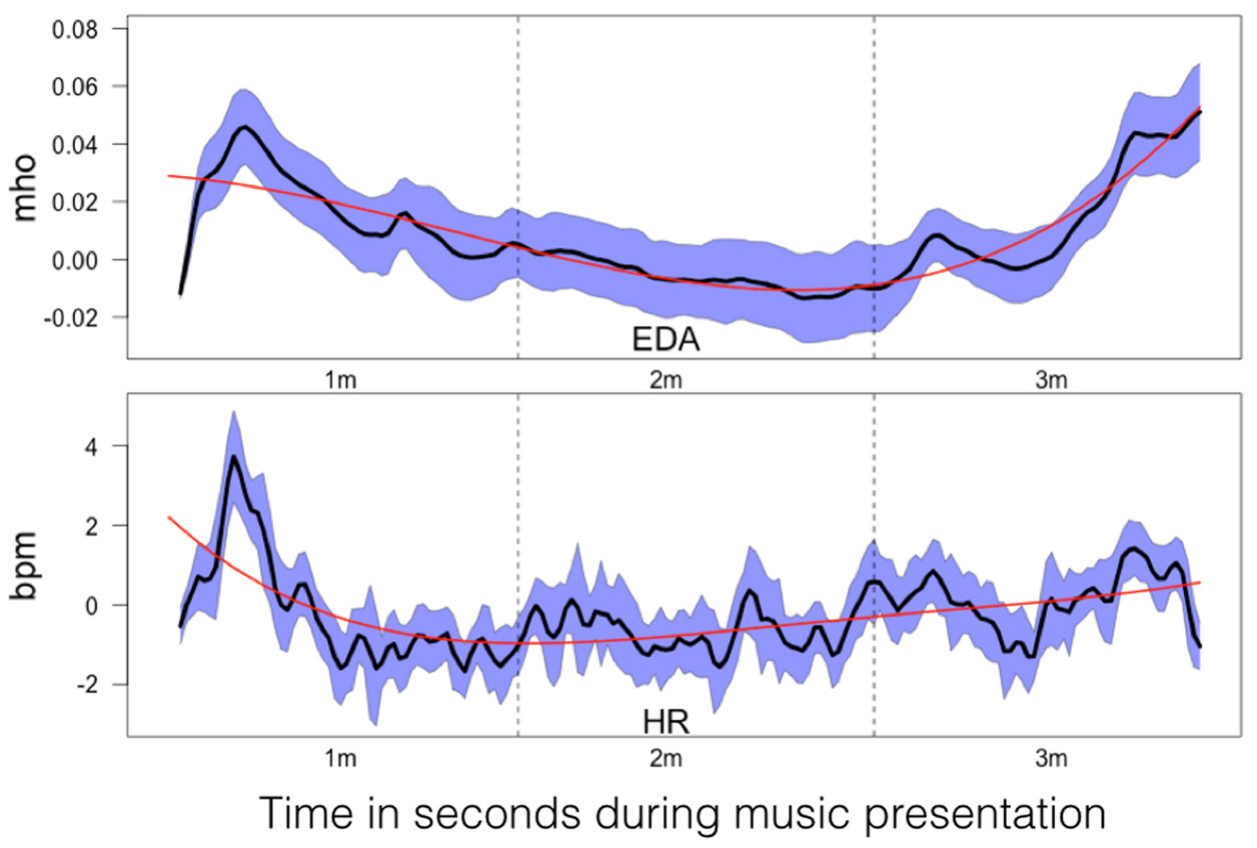
**Mean changes in electrodermal activity (EDA; in mho) and HR change (in bpm) relative to baseline during music listening.** The time scale is the same as for the time courses depicted in **Figure 8**. The dashed vertical lines separate the time courses into segments of 1 min duration (1m: 1st minute, 2m: 2nd minute, 3m: 3rd minute). The SEM (calculated across subjects) are plotted as blue area around the mean (plotted as a black line). The red line indicates the 4th order polynomials fitted to these time courses. These fits explain the time courses very well (EDA: *R*^2^ = 0.83; HR: *R*^2^ = 0.77).

The statistical analysis comprised several steps:

(1) Arousal and valence ratings obtained in Trial-1 and Trial-17 are compared using *t*-tests (*step 1*).
(2) We tested whether the EEG became synchronized (increased in terms of power) or desynchronized (decreased in terms of power) during the course of the music presentation. To do this, we calculated mean relative EEG measures for all EOIs and compared them to zero. For these tests, we performed no correction for multiple comparisons. Cohen’s *d*, with a threshold of *d* > 0.5, was used to decide whether the relative EEG measures deviated from zero (*step 2*).
(3) In order to examine whether the dynamics of the acoustic variables AMP and ACI are related to the dynamics of the EEG and psychophysiological measures, cross-correlations were done between the EEG time courses and the time courses of AMP and ACI. Here, we used different time resolutions ranging from a 1 Hz to a 0.028 Hz. For the high time resolution (1 Hz), we computed cross-correlations with different lags. However, since the correlations at lag = 0 turned out to be the strongest, we used these correlations for all of our analyses. For all correlation analyses, we computed the mean time courses separately for each subject across all repetitions (*step 3*). The results are shown in **Figures 5** and **6**.

(4) In a further step, we correlated (using the different time resolutions) the EEG time courses with the time courses of the psychophysiological variables (*step 4*). The results are shown in **Figures 5** and **6**.
(5) To examine whether the time courses of the EEG oscillations and the psychophysiological measures are stable or vary across the repeated presentations of the aria, we calculated between-trial correlations. Similar to the aforementioned correlations, we performed these correlations separately for the different time resolutions. In order to estimate the mean correlation of these between-trial correlations, we computed the “eigenvector” of the correlation matrix and identified the maximum and minimum eigenvalues and the corresponding range. By dividing the range of eigenvalues by 17 (the number of variables included in the eigenvalue analysis), we obtained the mean correlation of the correlation matrix. The correlations were then z-transformed and averaged across all subjects to obtain a mean “between-trial correlation” for all physiological variables. This “between-trial correlation” can be taken as a kind of reliability measure *(step 5)*. The results are shown in **Figure 7**.

(6) In order to describe the time courses, polynomials up to the fourth order were computed for all time courses *(step 6)*. The results are shown in **Figures 8** and **9**.

(7) All the above-mentioned analyses (except for *step 1*) were done on a descriptive basis. For statistical testing, we calculated a series of ANOVAs. First, we performed a four-way repeated measures ANOVA with *Trial* (17 levels; Trial-1–Trial-17), *Time Frame* (three levels: 1st, 2nd, and 3rd minutes of the musical piece), *Scalp Location* (three levels: frontal, central, and parietal) and *Site* (three levels: left, central, and right) for all relative EEG measures. For these analyses, we computed the mean relative EEG values for the 1st, 2nd, and 3rd minutes of the musical piece. Since we used five frequency bands, we performed five four-way repeated measures ANOVAs *(step 7)*.
(8) Two-way repeated measures ANOVA was performed for EDA and HR, using *Trial* (17 levels; Trial-1–Trial-17) and *Time Frame* as independent variables (three levels: 1st, 2nd, and 3rd minutes of the musical piece) (*step 8*).
(9) In order to test for differences between frequency bands (*Frequency*) and interactions between *Scalp Location* (frontal, central, and parietal), *Site* (left, central, and right), and *Time Frame* (1st, 2nd, and 3rd minutes), we conducted a four-way repeated measures ANOVA with the relative EEG measures as the dependent variables (s*tep 9*). The results are shown in **Table 2**.

The analyses done in *steps 2–6* were entirely descriptive. In these analyses, we calculated correlation coefficients or Cohen’s *d*, categorized as proposed by Cohen (1992), where 0.3 < *r* < 0.5 is considered to be a moderately strong correlation. In this context, a correlation of *r* > 0.5 is a strong correlation. For the statistical analysis of the rating data (*step 1*), we conducted several *t*-tests for which we applied the Bonferroni–Holm adjustment, starting with *p* = 0.05. For *d*, we also relied on the classification provided by Cohen (1992; moderate effect: 0.5 < *d* < 0.8, large effect: *d* > 0.8). For the ANOVAs performed in *steps 7–9*, we used the Greenhouse–Geisser correction to estimate the *p*-values. Since *p*-values are more or less uninformative when calculated on the basis of a relatively small sample, as we have used here, we also focused on effect size measures. Thus, we report $\eta_{p}^{2}$ effect size measures, as provided in the SPSS output. Since we performed several ANOVAs (step 7: *n* = 5; step 8: *n* = 2; step 9: *n* = 1; total: *n* = 8), it was necessary to correct for multiple statistical tests. Here, we used the Bonferroni–Holm correction starting with *p* = 0.00625 (=0.05/8). For all statistical analyses, SPSS for Mac (Version 22) and several R packages were used. R was used for data handling, sorting, correlation analyses, and plots.

## Results

### Comparisons of Subjective Rating (Step1)

The mean valence and arousal ratings obtained after Trial-1 and Trial-17 are displayed in **Table 1**. In general, the musical piece was rated as positive and arousing. However, the valence and arousal ratings dropped following the last trial [valence: *t*(15) = 3.0, *p* = 0.008; arousal: *t*(15) = 2.6, *p* = 0.021].

**Table 1:** Summary of valence and arousal ratings obtained after Trial-1 and Trial-17.

		Trial-1	Trial-17	*t* (*df* = 15)	*p*
**Valence**					
	*m*	472	184	3.0	0.008
	SD	240	338		
**Arousal**					
	*m*	601	415	2.6	0.021
	SD	193	245		

*Please note that the scale ranged from -1000 (unhappy) to +1000 (happy) for valence and from 0 (not aroused) to 1000 (excited) for arousal. m, mean; SD, standard deviation; t, t-value, p, two-tailed p-value, df, degrees of freedom.*

### Two-Way ANOVAs for the Psychophysiological Measures (Step 8)

The two-way repeated measures ANOVA for EDA and HR revealed strong effects for Time Frame but no influence of Trial, either as main effect or as part of the interaction with Time Frame [EDA: *F*(1.1,16.6) = 19.6, *p* < 0.001, η^2^ = 0.57; HR: *F*(1.26,18.9) = 11.7, *p* = 0.002, η^2^ = 0.44]. Subsequently performed *post hoc* tests (Bonferroni–Holm corrected) revealed that the EDA and HR measures were small during the 2nd minute, with no difference between the 1st and 3rd minutes.

### Examination of Increases or Decreases in Relative EEG Measures (Step 2)

The relative EEG measures were all positive, indicating increased power relative to baseline. We averaged these measures across all trials and used Cohen’s *d* to evaluate whether they deviated considerably from zero. All relative EEG measures deviated considerably from zero (as determined by *d* > 0.6), indicating that the relative power in all frequency bands becomes larger during music listening. Thus, all EEG measures demonstrated ERS. The weakest ERS was found for beta power during the 1st minute of the music presentation, although it did pass the threshold for a large effect (mean = 0.06, SD = 0.10, *d* = 0.6). All further effect sizes were much larger, demonstrating strong power increases for all frequency bands.

### Correlations between the Time Courses of the Acoustic and Physiological Variables (Steps 3 and 4)

In order to identify similarities between the time courses of the acoustic and physiological variables, we performed correlations between variables. These correlations were done for different time resolutions [175 time frames (period of 1 s) = 1 Hz, 35 time frames (period of 5 s) = 0.2 Hz, 25 time frames (period of 7 s) = 0.14 Hz, 7 time frames (period of 25 s) = 0.04 Hz, 5 time frames (period of 35 s) = 0.028 Hz]. For the 1 Hz time resolution, correlations were done using lags of up to 10 s. The correlations for lag = 0 turned out to be the strongest correlations, and we thus used them for our analyses. All correlations were performed separately for each subject, transformed using Fisher’s z transformation, averaged, and then re-transformed from z-transformed correlations to regular correlations. These correlations are depicted in **Figure 5** (upper). As can be seen in **Figure 5**, the correlations with the acoustic variables were small and far from substantially large (e.g., *r* > 0.5). Furthermore, the correlations increased with decreasing time resolution and we obtained the strongest correlations for the lowest time resolutions. For example, beta correlated quite strongly with AMP and ACI: the stronger AMP and ACI, the stronger the beta power synchronization. A similar picture emerged for all other frequency bands. For the psychophysiological variables, we found stronger negative correlations with the acoustic variables. Thus, with increasing acoustic complexity, HR and EDA became smaller.

For the correlations between the psychophysiological and neurophysiological variables, we obtained similar results (**Figure 5**, lower). With a high time resolution, the correlations were small. For the correlations obtained for the time series with low resolution, the correlations were much higher. These larger correlations were negative, indicating that as ERS increases, HR and EDA decrease.

**Figure 6** gives the correlations between the EEG measures. All correlations were small or moderate. In general, the correlations increased with diminishing time resolution. For the lowest time resolution, we obtained the largest correlations between all EEG measures, with several strong correlations, according to Cohen’s classification.

### Correlations between the Time Courses Obtained for Repeated Presentations of the Musical Stimulus (Step 5)

**Figure 7** shows the correlations between the time courses obtained during the 17 trials. As can be seen from the **Figure 7**, the correlations (which can be interpreted as reliability indices) are low for the high time resolutions. For the high time resolutions, moderately strong stability is only seen for the EDA time course. The EEG time course measures are more stable across the 17 repetitions for the low time resolutions. However, for the lowest time resolution, we obtained only moderately strong correlations. The EDA time course turned out to be the most stable time course across all repetitions. For the high time resolutions, the mean correlations were moderately strong.

### Analysis of Time Courses (Step 6)

The mean time courses for all variables are depicted in **Figure 8**. For the ERS time courses, we used the central EOIs, since these time courses exhibited the strongest variation within music presentation. In **Figure 8**, the time courses are depicted together with the SEM and 4th order polynomials. The 4th order polynomials explain the mean time courses quite well (theta: *R*^2^ = 0.53; low-alpha-1: *R*^2^ = 0.61; low-alpha-2: *R*^2^ = 0.77; upper alpha: *R*^2^ = 0.70; beta: *R*^2^ = 0.78; GFP: *R*^2^ = 0.53). As can be seen in the time courses, sharp increases in ERS occurred during the 1st minute of music presentation for theta, low alpha-1, low alpha-2, upper alpha, and for the GFP. During the following 2 min of music, ERS was more or less stable. For beta, a more or less stable linear increase occurred throughout the 1st and 2nd minutes of the music presentation. During the final minute, no substantial change in beta ERS was seen. GFP is included here since this measure represents the strength of the topographic map. Thus, the larger the GFP, the stronger the difference between the positive and negative amplitudes of the EEG and the more pronounced the difference between the smallest and the largest ERS over the entire electrode set.

The mean time courses for the psychophysiological variables are shown in **Figure 9**. These time courses can be explained by 4th order polynomials (EDA: *R*^2^ = 0.83; HR: *R*^2^ = 0.77). Both time courses are roughly similar, with an initial drop occurring within the 1st minute. EDA increased during the final minute, while HR remained more or less constant throughout the music presentation.

### Four-Way ANOVAs for the Neurophysiological Measures (Step 7)

The four-way repeated measures ANOVAs (Trial: 1–17, Time Frame: 1–3, Scalp Location: frontal, central, and parietal, Site: left, mid, right) for the ERS measures revealed no significant main effect for Trial or any interaction between Trial and other factors. There were, however, strong and significant results for Time Frame for all frequency bands [theta: *F*(1.5,22.7) = 9.4, *p* = 0.002, η^2^ = 0.38; low alpha-1: *F*(1.2,18.3) = 11.5, *p* = 0.002, η^2^ = 0.43; low alpha-2: *F*(1.5,23.5) = 22.4, *p* ≤ 0.001, η^2^ = 0.59; upper alpha: *F*(1.6,24.5) = 24.3, *p* < 0.001, η^2^ = 0.62; beta: *F*(1.7,19.1) = 26.8, *p* < 0.001, η^2^ = 0.64]. Subsequently performed *post hoc* tests (Bonferroni–Holm corrected) revealed that the ERS values were stronger during the 2nd and 3rd minutes of the music presentation than in the 1st minute. For most EEG measures, there were no differences in ERS during the 2nd and 3rd minutes of the music presentation, except for the beta band, where there was a linear increase from the 1st to the 3rd minutes (3 > 2, 2 > 1). For theta and all alpha bands, significant effects were seen for Scalp Location and Site. Bonferroni–Holm corrected *post hoc* tests revealed that the ERS values at the central and parietal sensor positions were the strongest, compared to the frontal positions. Site turned out to be significant for theta, low alpha-1, and low alpha-2, with the central sensor position yielding the largest ERS measures. Comparing ERS values obtained from the left and right sensors revealed practically identical measures for all frequency bands, except for low alpha-2, for which the right-sided sensors yielded the strongest ERS measures (*p* = 0.059, corrected for multiple comparisons).

### Four-Way ANOVA for Testing between Different Frequency Bands (Step 9)

**FIGURE 10:**
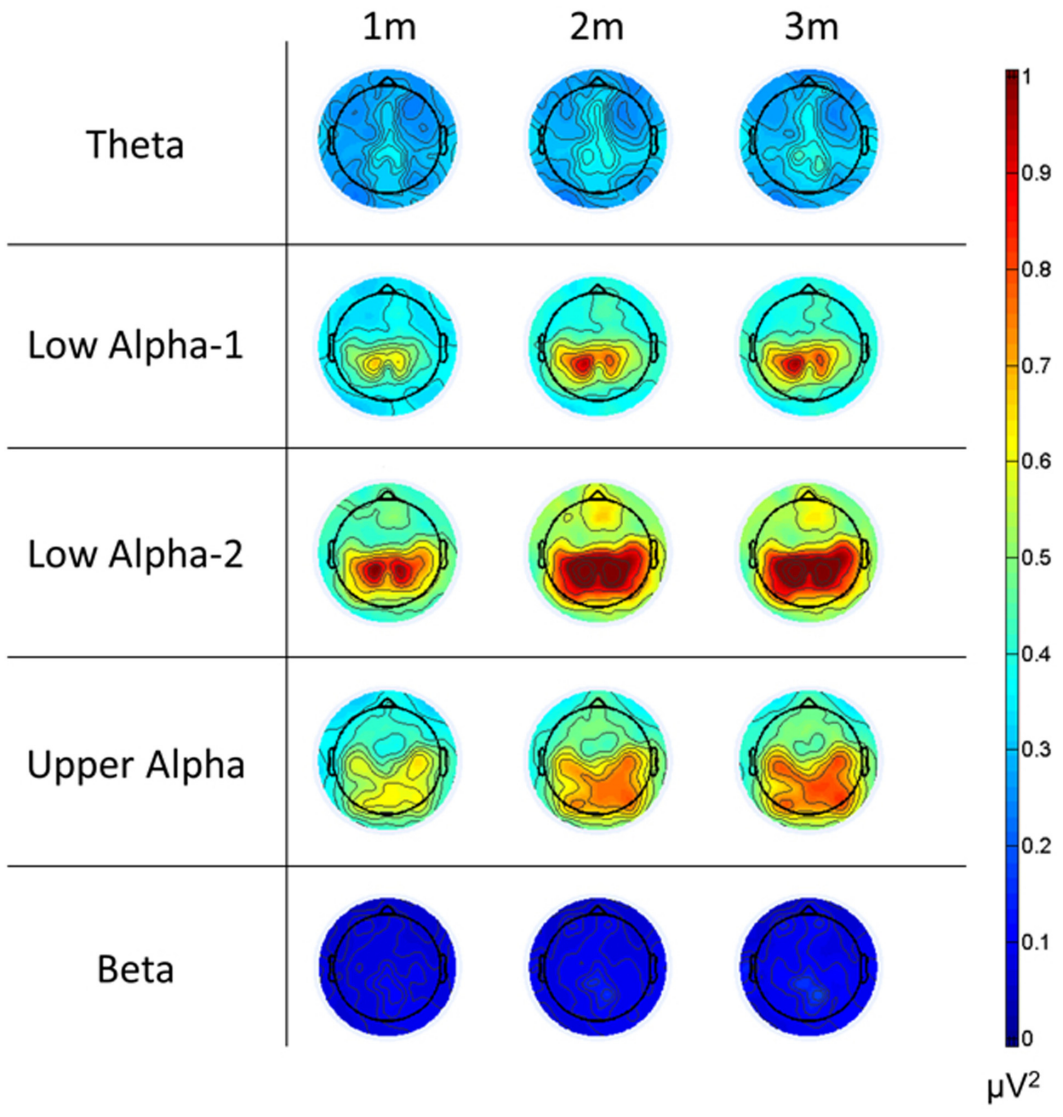
**Topoplots of the relative EEG power changes relative to the baseline for each frequency band and for the 3 min of the music presentation.** Please note that we used the same scale for all frequency bands. All changes in power are positive, indicating ERS. (1m: 1st minute, 2m: 2nd minute, 3m: 3rd minute).

**FIGURE 11:**
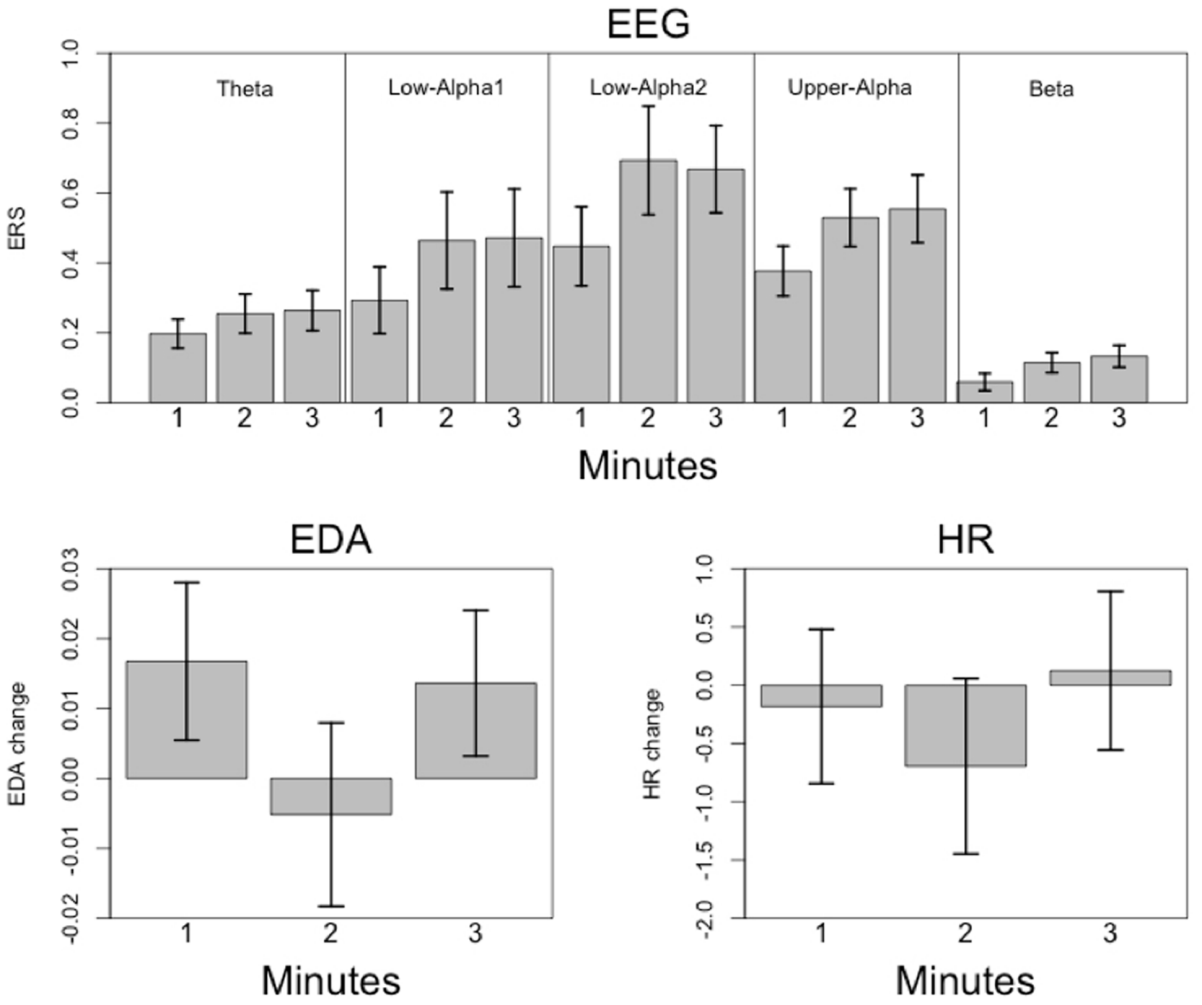
**Mean relative EEG power changes (and SEM; **Upper**) and mean **psychophysiological** measures **(Lower)** for each minute of music listening (1m: 1st minute, 2m: 2nd minute, 3m: 3rd minute)**.

Since Trial turned out to be insensitive, we calculated mean values for ERS and the psychophysiological measures and subjected these measures to a four-way repeated measures ANOVA with Time Frame (1st, 2nd, and 3rd minutes of music listening), Site (left, central, and right), Scalp Location (frontal, central, and parietal), and Frequency (theta, low alpha-1, low alpha-2, upper alpha, and beta) as independent variables. The significant effects are shown in **Table 2**. The main effect of Frequency is qualified by larger ERS for low alpha-2 and upper alpha. The smallest ERS was found for beta and theta, with no differences between them. No differences were seen between low alpha-1 and upper alpha, low alpha-1 and theta, or low alpha-2 and upper alpha. Low alpha-2 did differ, however, from low alpha-1. The main effect of Scalp Location depended on larger ERS for central and parietal EOIs. The main effect of Site is qualified by large ERS over central and parietal EOIs. The smallest ERS was found at the frontal EOIs. The main effect of Time Frame depended on the fact that the ERS seen during the 1st minute is smaller than the ERS observed during the 2nd and 3rd minutes. Five two-way interactions were also significant: Scalp Location × Time Frame, Frequency × Time Frame, Frequency × Scalp Location, Frequency × Site, and Time Frame × Site. No further interaction became significant. Scalp Location × Time Frame is qualified by higher ERS found for the central and parietal EOIs, with no difference between the central and parietal EOIs. Frequency × Time Frame depended on differences in the power increase from the 1st to the 2nd and 3rd minutes of the music presentation. Descriptively, the strongest power increases were for low alpha-1 (mean = 0.16) and low alpha-2 (mean = 0.16), followed by upper-alpha (mean = 0.095). The smallest increases were found for beta (0.04) and theta (0.057). Bonferroni–Holm corrected *post hoc* tests revealed no significant differences between beta and theta, beta and low alpha-1, low alpha-1 and upper alpha, or low alpha-2 and upper alpha. However, low alpha-2 showed stronger increases from the 1st to the last 2 min, compared to theta, beta, and upper alpha. In addition, upper alpha revealed stronger increases than theta and beta. The Frequency × Scalp Location interaction was determined by the fact that there is no significant difference across frontal, central, and parietal EOIs for the beta band. For all other frequency bands, the central and parietal EOIs demonstrated stronger power increases than did the frontal EOI. Frequency × Site is qualified by larger power increases of the central EOI for low alpha-1 and low alpha-2. For the other frequency bands, we found no differences between the left, central, and right EOIs. The final significant interaction is qualified by larger increases from the 1st minute to the last 2 min over central EOIs. No differences were seen with respect to power increases of left or right EOIs (central: mean = 0.14, SD = 0.10; left: mean = 0.11, SD = 0.07; right: mean = 0.12, SD = 0.08). The mean relative EEG measures for each frequency band are shown as topoplots in **Figure 10**. In addition, the mean ERS are shown in **Figure 11**, together with the mean HR and EDA measures.

**Table 2:** Significant results for four-way repeated measures ANOVA with Frequency, Time Frame, Scalp Location, and Site as the independent variables.

Effect	*F*	*df1*	*df2*	*p*	η^2^
Frequency	14.30	1.60	25.10	0.001	0.49
Time Frame	11.70	1.70	25.70	0.001	0.44
Scalp Location	25.30	1.50	22.40	0.001	0.63
Site	6.30	1.20	18.10	0.001	0.24
Frequency × Time Frame	8.80	2.50	38.10	0.001	0.37
Frequency × Scalp Location	4.80	4.10	61.40	0.001	0.24
Frequency × Site	4.61	2.50	37.57	0.010	0.24
Time Frame × Site	3.39	2.40	35.60	0.040	0.18
Time Frame × Scalp Location	6.80	2.16	32.36	0.001	0.31

*The dependent variables are the event-related synchronizations (ERS) for all frequency bands (theta, low alpha-1, low alpha-2, upper alpha, and beta). Only those effects passing a significance level *p* < 0.05 are shown. df1 and df2 are the Greenhouse–Geisser corrected degrees of freedom, *p* is the *p*-value, and η^2^ is the $\eta_{p}^{2}$ provided in the SPSS output.*

## Discussion

This study was designed to examine five questions: (1) Does a consistent relationship exist between the time course of EEG oscillations and the time course of particular acoustic features of the presented musical piece? (2) Similarly, are the time courses of psychophysiological measures (HR and EDA) related to the time courses of the acoustic features of the musical piece? (3) How stable are the time courses of EEG oscillations and the time courses of psychophysiological measures across repeated music presentations? (4) Do the neurophysiological responses that occur during music listening change across repeated presentations of the same musical piece? (5) Similarly, do EDA and HR measured during music listening change across repeated exposures to the same musical piece? For the musical piece, we chose a popular and well-known aria lasting more than 3 min. We chose this aria because its acoustic intensity varies considerably throughout the 3 min. The aria was presented 17 times to the subjects, while EEG oscillations, HR, and EDA were continuously measured.

So far, examination of the time course of EEG oscillations during music listening has rarely been done. Two recent studies examined neurophysiological responses to realistic musical pieces (Cong et al., 2013; Sturm et al., 2014), but the results are difficult to relate to our project. The study by Cong et al. (2013) was published in the proceedings of a technical conference and clearly focused on the elaboration of specific techniques for analyzing the time courses of EEG data, without reference to emotional responses or repeated presentation musical stimuli. The paper by Sturm et al. (2014) reports on ten epileptic patients from whom the investigators measured electrocorticographic (ECoG) signals from 53 to 134 channels placed only over the left perisylvian brain while the patients listened to a rock song and a narrative. This was an invasive manipulation, thus definitively demolishing the pleasureful aspect of listening to music.

Our study does, however, have a strong relation with the two studies of Mikutta et al. (2012, 2014), which specifically examined the time courses of EEG oscillations and emotional responses during music listening. In both studies, rather than studying the time courses of EEG oscillations in detail, the authors used an approach they named “covariance mapping.” In this technique, the correlations (or covariances) between the time courses of subjective ratings (here, arousal) and the EEG oscillations are calculated and mapped onto topoplots. In this context, the particular time courses are not analyzed in detail. In addition, the authors of this pioneering work related the time courses of subjective ratings obtained in separate sessions to the EEG time courses. Thus, it is not clear whether the sequential measurement might have influenced their results. In our study, we examined the EEG oscillation time courses in more detail and related them to the time courses of acoustic features and psychophysiological variables. EDA and HR were used as psychophysiological measures representing general arousal, thus providing the opportunity to simultaneously obtain arousal measures with the EEG oscillations. Since no published studies have looked at the stability of EEG oscillations in the context of repeated presentation of musical pieces, we presented 17 repetitions of our chosen musical piece. This frequent repetition may mimic the repeated listening of musical pieces done in real-life situations. Furthermore, a wealth of studies have shown that our physiological system adapts, or habituates, to repeated exposure to the same stimulus. In the following section, we will discuss our results in a more comprehensive manner.

### Behavioral and Psychophysiological Data

While the initial valence and arousal ratings indicate a general rating of the aria as positive and as arousing, different ratings were obtained after the 17th presentation of the aria. After the final presentation, the subjects rated the aria as less arousing and less positive than after the first presentation. Thus, a kind of habituation in terms of subjective rating took place. Habituation is normally accompanied by typical changes in psychophysiological measures, e.g., reduced HR and EDA reactions (Holzl et al., 1975; Elton et al., 1983). However, we did not find a habituation-like reduction in psychophysiological responses after repeated presentations. The HR and EDA responses to the musical stimuli were roughly similar across the 17 presentations. Thus, subjective ratings and psychophysiological responses do not correlate with one other and actually dissociate. Inspecting the mean HR time course reveals a pattern of initial acceleration up to 4 bpm (lasting ∼10 s) followed by HR deceleration within the 1st minute of music presentation. During the final 2 min of the music presentation, HR normalizes to the baseline level. A similar time course was found for EDA, which showed an initial increase followed by a decrease. However, at the end of the aria presentation, when the intensity of the acoustic stimulus increased, EDA responses increased again. Since EDA responses are known to correlate with subjective arousal (Ellis and Simons, 2005), it is likely that the louder music at the end of the presentation evoked greater psychophysiological arousal. A roughly similar HR time course in response to positively rated musical pieces was reported by Sammler et al. (2007), who saw an initial decrease in HR in the first 2 s of music presentation, followed by HR acceleration and normalization to baseline levels after 4–6 s. Our findings demonstrate that the chosen musical stimulus induces pleasant emotions and arousal in listeners. They also demonstrate that valence and arousal ratings, but not psychophysiological measures, change during repeated presentation. The psychophysiological measures reflect a typical pattern that is relatively stable during the presentation of the musical stimuli (see **Figure 7**). In addition, the HR and EDA responses to our musical stimulus replicate the findings reported by similar studies (Ellis and Simons, 2005; Sammler et al., 2007).

### EEG Oscillations

For the EEG oscillations, we observed increases in power (relative to baseline) for all frequency bands throughout the music presentation. The strongest increases were found during the 2nd and 3rd minutes of the music presentation, especially for the alpha bands (low-alpha-1, low-alpha-2, and upper alpha). ERS for all frequency bands was strongest at the central and parietal EOIs, with no left-right differences observed. Interestingly, no consistent changes in EEG oscillations were seen during the 17 trials of repeated music presentation. This does not mean that the EEG oscillations were constant during the 17 repeated presentations, but that they were variable, as shown by the weak inter-trial correlations between the EEG time courses (**Figure 7**). In addition, we found no consistent patterns of change that might indicate habituation, increased alertness, or drowsiness. Furthermore, the time courses of the psychophysiological measures did not strongly correlate with the time courses of EEG oscillations, suggesting a kind of dissociation between psychophysiological and neurophysiological activation (**Figure 5**). This lack of a strong correlation between psychophysiological measures and EEG oscillations is especially apparent for the high time resolutions (1 and 0.2 Hz). Lower time resolutions for the physiological measures (0.14 and 0.028 Hz) were associated with considerably stronger correlations between the psychophysiological and neurophysiological measures, with moderately strong correlation between the psychophysiological measures (EDA and HR) and low-alpha-1, low alpha-2, and upper alpha.

Although the general pattern of ERS across music presentations was similar for all frequency bands, substantial differences were also found, as indicated by the weak to moderate correlations between EEG oscillation time courses (**Figure 6**). Thus, the different frequency bands reflect different neurophysiological or psychological mechanisms being activated during music listening. In the following section, we will discuss what the findings for the different frequency bands might indicate. We will also provide an overarching interpretation of the neurophysiological and psychological processes most likely to be involved when listening to our particular musical piece.

#### Theta Band

Increases in human theta oscillations obtained through scalp EEG are largely associated with three different psychological functions: (a) the so-called frontal midline theta (Fm theta), which is generally related to cognitive effort, working memory, error, or processing of emotion (Gevins et al., 1997; Gevins and Smith, 2000; Onton et al., 2005; Kawamata et al., 2007; Sammler et al., 2007; Michels et al., 2008; Lin et al., 2010; Anguera et al., 2013; Enriquez-Geppert et al., 2014; Maurer et al., 2015; Wisniewski et al., 2015); (b) the widespread theta most prominent at the frontal and parietal scalp locations, which is associated with low-level alertness, drowsiness, and “mind-wandering” (Braboszcz and Delorme, 2011; Park et al., 2011; Platt and Riedel, 2011; Baumeister et al., 2012; Poudel et al., 2014), and (c) the widespread theta with parietal dominance, which has been related to effective encoding of new memories (Klimesch, 1999). Theta increases have also been reported during meditation (Kasamatsu and Hirai, 1966; Wallace et al., 1971; Banquet, 1973; Lagopoulos et al., 2009; Baijal and Srinivasan, 2010; Faber et al., 2012).

The theta ERS found in our study was more the widespread theta type, although the strongest theta ERS was found at fronto-central midline positions, which might be with Fm theta at least at that location. In line with Sammler et al. (2007) and Lin et al. (2010), one could argue that our subjects were highly emotionally engaged during the music listening. Fm theta is driven mainly by dipoles located either in the anterior cingulum (ACC) or the medial frontal cortex, brain areas that are both strongly involved in processing of emotion via fronto-striatal and fronto-limbic loops (Gevins et al., 2012). Thus, the more strongly these loops are activated, the stronger the Fm theta. A further possibility could be that our subjects were literally ‘drawn into’ the music, which would result in less attention paid to the outside world. The subjects would then have started a psychological process that could be similar to “mind-wandering,” a state during which theta increases have been frequently reported (Braboszcz and Delorme, 2011). Mind wandering is a phenomenon that occurs quite often in everyday life. It is characterized by the experience of one’s attention drifting away from a task or from outside matters toward personal issues. Braboszcz and Delorme (2011) argue that mind wandering is a sort of low-alertness and low concentration state of rest. It can also be seen as a hypnagogic state similar to the psychological and neurophysiological states identified during meditation. It should be noted that being in a mind-wandering state does not prevent emotional experience. Thus, it is conceivable that mind wandering during music listening is still associated with activations in the fronto-striatal and fronto-limbic systems driving emotional experience and thus Fm theta.

#### Alpha

Electroencephalography alpha power increases have been consistently reported during psychological states not requiring attention to the environment, that is, during internally directed attention (Cooper et al., 2003; Palva and Palva, 2007). A typical example is the resting-state during which subjects are not performing explicit tasks. During task-free resting-state situations, spontaneous fluctuations in alpha oscillation power correlate negatively with activity in the so-called dorsal attention system (DAT) related to the superior frontal and intraparietal regions (Laufs et al., 2003). Thus, when the dorsal attention system is deactivated (because the subject is not using externally directed attention) alpha power increases. There is, however, a positive correlation between spontaneous fluctuations in alpha oscillation power and activity in a more ventrally located cingulo-opercular (CO) network comprised of the dorsal anterior cingulate cortex, frontal operculum/anterior insula, and thalamus, all brain areas that have been related to sustained cognitive control and maintenance of alertness (Dosenbach et al., 2007). Taken together, these findings show that increased alpha power, mostly low alpha-2 and upper-alpha, is associated with decreased activation in the dorsal attention system (indicating reduced external attention) and with increased activation in the ventral CO network (indicating sustained alertness). Thus, there is a growing consensus that increased alpha power is associated with active inhibition of sensory networks or brain networks that are not needed for the control on-going tasks but rather for simultaneous maintenance of alertness (Sadaghiani et al., 2009; Coste et al., 2011; Sadaghiani and Kleinschmidt, 2013; Schiff et al., 2014).

This also fits with the studies reporting increased alpha power, mostly at centro-parietal sites, during working memory tasks (Klimesch, 1999; Klimesch et al., 1999; Palva and Palva, 2007; Weisz et al., 2011) or during effortful cognition such as perception of degraded speech (Weisz et al., 2011). In such situations, the engaged neural networks need to maintain optimal neural activation by maintaining an optimal level of excitation–inhibition through suppression of neural network synchronization, which might “disturb” or “interfere” with the on-going processing of the relevant task. Thus, it is not surprising that increases in alpha power are also seen during meditation, which is typically associated with the redirecting of attention from external events to internal thoughts (Aftanas and Golocheikine, 2001; Faber et al., 2012).

Increased alpha power has also been related to emotional processing (Aftanas et al., 2001, 2006; Yuvaraj et al., 2014a,b). More importantly, a considerable body of EEG research has focused on the relationship between emotional processing and frontal alpha power asymmetry, leading to the formulation of the so-called “hemispheric valence hypothesis” (Davidson, 1998). This hypothesis states that positive emotions are processed mainly in left frontal brain areas, whereas negative emotions engage the right frontal brain regions. This difference in hemispheric activation is thought to be associated with an alpha power asymmetry, especially at the frontal scalp positions. Thus, a decrease in left frontal alpha power should be evident during processing of positive emotions and a decrease in right frontal alpha power should be seen during processing of negative emotions. A number of studies using different types of emotional stimuli (e.g., film clips, reward or punishment, or pictures) have supported this hypothesis (Davidson et al., 1990; Sobotka et al., 1992; Aftanas et al., 2001; see also Hagemann et al., 1998; Reid et al., 1998; Hagemann, 2004; Baumgartner et al., 2006a; Sammler et al., 2007). Two EEG studies using emotional music have also supported this frontal asymmetry hypothesis (Schmidt and Trainor, 2001; Tsang et al., 2001). In addition, although they used DC potentials and not alpha band power, Altenmüller et al. (2002) reported a frontal brain asymmetry for pleasant and unpleasant music and sounds that favors the frontal asymmetry hypothesis. However, in our study, we found no strong evidence for a frontal alpha asymmetry, except for the low-alpha-2 band, which showed a slightly and non-significantly stronger ERS at the right-sided frontal EOIs. A recent study Mikutta et al. (2012) reported a right-frontal suppression of lower alpha-band activity during high subjective arousal while the subjects listened to a musical piece. However, the study did not relate the alpha power to emotional ratings. In sum, a direct and clear influence of emotional processing on alpha power oscillations is not obvious from the current body of knowledge. Nevertheless, alpha power appears to be more strongly related to active inhibition of sensory or brain networks that are not needed for control of an on-going task.

#### Beta

Electroencephalography beta frequency oscillations generally indicate activation, excitation, and facilitation (Lopes da Silva, 1991). They increase during attention (Wrobel, 2000; Kisley and Cornwell, 2006; Basile et al., 2010) and with alertness (Oron-Gilad et al., 2008; Hlinka et al., 2010; Gola et al., 2012; Kaminski et al., 2012). A few studies have found an association between beta power and emotional processing, with most reporting an increase in beta power following an unspecific increase in emotional arousal (Sebastiani et al., 2003; Aftanas et al., 2006). Apart from the above-mentioned findings, a circumscribed, centrally distributed cortical beta rhythm is strongly related to motor function and has been found to originate from the primary motor cortex (Pfurtscheller and Lopes da Silva, 1999; Oron-Gilad et al., 2008; Bauer et al., 2014) as well as from the supplementary motor area (Pfurtscheller et al., 2003). However, increases in beta oscillations have also been observed during the “minimally conscious state” (MCS; Schiff et al., 2014), a finding that seems to be, at least at a first glance, somewhat counterintuitive according to the aforementioned findings. For the MCS, it is suggested that the often-seen increase in theta and beta oscillations could be a consequence of a kind of functional or structural deafferentation of the thalamus from its cortical inputs, which should finally result in theta and beta bursts. The relative beta power increases seen in our study were small compared to the power increases found for the other frequency bands. Although small, these increases became stronger during the course of music listening, with the strongest increases found at centro-parietal EOIs. Whether these increases were due to emotional processing, increased alertness, a psychological function similar to MCS, or a combination of these processes, is unclear at present.

## Conclusion

The general EEG response pattern seen in our study is characterized by a general power increase relative to baseline for all frequency bands. Such a neurophysiological response pattern is unusual and has rarely been reported to date. Interestingly, the time courses of the EEG oscillations did not correlate strongly with one other, and thus one cannot argue that the oscillations depended on a kind of fundamental oscillation driving and influencing all other oscillations. The EEG time courses were partially independent from one other. Obviously, the different oscillations indicate different neurophysiological and psychological processes that are operative while listening to the particular musical piece. The small correlations between the EEG time courses within and between trials indicate strong variability in these neurophysiological and psychological processes during music listening. Nevertheless, there is a general neurophysiological and psychological state that is obviously dominant during music listening. We speculate that this state is characterized by increased internal attention, accompanied by reduced external attention, increased inhibition of brain networks uninvolved in generating this internal state (thus maintaining a particular level of general alertness) and a mind-wandering state. The overall state fits with a psychological process in which the listeners are ‘drawn into’ the musical piece and become even more drawn into the music the longer they listen to the piece.

### Limitations

A number of limitations are associated with this study. Besides the fact that the study used only 16 subjects (a sample size frequently used in studies of this type but which is nevertheless relatively small), the variability in the EEG time courses is remarkable. As can be seen from **Figures 5–7**, the variability within and between the EEG frequency bands decreases with lower time resolution. In our opinion, this does not indicate a technical measurement problem but rather the variability in the sequence of psychological and physiological processes operating during music listening. It is possible that the time course of EEG activity is composed of relatively short-lasting brain states varying over time. For example, Lehmann et al. (1987) showed that continuous EEG recordings can be parsed into a series of distinct “microstates” that are defined as time periods that remain stable in the sub-second time-range and that are separated by rapid configuration changes. In a recent review on that topic, Koenig et al. (2002) demonstrated that the duration of these microstates ranges between 80 and 120 ms, a duration which roughly matches the minimal duration of a percept. Lehmann (1990) thus proposed that microstates might correspond to basic blocks of human information processing. Microstates have been shown to depend on “what just went through the mind” (Lehmann et al., 1998). Thus, it is likely that the sequence of thoughts and feelings might vary from subject to subject, but also intra-individually during music listening. That could explain the variability in the EEG time courses obtained with high temporal resolution. The EEG time courses with low time resolution thus might represent a more general change in neurophysiological activation associated with a more general and slower change in psychological state. We suggest that the slower and more general changes occurring during music listening reflect being more ‘drawn into’ the musical piece the longer the music lasts. The faster neurophysiological and psychological processes will be studied by our group in future studies, where we will use different techniques to analyze our EEG data. Here, we have focused on the EEG oscillations.

A further point that we would like to address as a limitation is the fact that we did not control the “listening style” or “listening attitude” applied by the subjects, a problem that is associated with nearly all studies examining the neural underpinnings of music listening. When listening to music, we often use different listening attitudes (e.g., emotional or cognitive listening) depending on our current mood or the motivation for listening to particular musical pieces. These listening attitudes might have changed within the subjects during repeated exposure, and the different subjects might have differed in terms of their use of these listening styles.

However, despite these limitations, this study has found that the neurophysiological activations during music listening are not stationary, but rather are dynamic. We have also demonstrated that the dynamics of these neurophysiological activations are not related to particular acoustic features. The psychological functions associated with these dynamic changes are a subject for future experiments.

## Conflict of Interest Statement

The authors declare that the research was conducted in the absence of any commercial or financial relationships that could be construed as a potential conflict of interest.

## Footnotes

1 http://www.esseraudio.com/de/home-audiometer-hoertest.html

2 http://sccn.ucsd.edu/eeglab/plugins/ASR.pdf

3 http://sccn.ucsd.edu/eeglab/plugins/clean_rawdata0_31.zip
